# Retraction: The role of ICT investment, digital financial inclusion, and environmental tax in promoting sustainable energy development in the MENA region: Evidences with Dynamic Common Correlated Effects (DCE) and instrumental variable-adjusted DCE

**DOI:** 10.1371/journal.pone.0323720

**Published:** 2025-05-07

**Authors:** The PLOS One Editors

The *PLOS One* Editors retract this article [1] due to concerns about potential manipulation of the publication process. These concerns call into question the validity and provenance of the reported results. We regret that the issues were not identified prior to the article’s publication.

In addition, please note that References 61, 73, 107, 128, and 131 were retracted after this article [1] was published.

MQ did not agree with the retraction. LX either did not respond directly or could not be reached.
